# Differential Expression Patterns of Rspondin Family and Leucine-Rich Repeat-Containing G-Protein Coupled Receptors in Chondrocytes and Osteoblasts

**DOI:** 10.22074/cellj.2021.6927

**Published:** 2020-04-22

**Authors:** Yeon-Hee Lee, Ashish Ranjan Sharma, Supriya Jagga, Sang-Soo Lee, Ju-Suk Nam

**Affiliations:** Institute for Skeletal Aging and Orthopedic Surgery, Hallym University-Chuncheon Sacred Heart Hospital, Chuncheon, Korea

**Keywords:** Chondrocyte, LGR, Osteoarthritis, Osteoblast, R-spondin

## Abstract

**Objective:**

Rspondins (RSPOs) are regarded as the significant modulators of WNT signaling pathway and they are expressed dynamically during developmental stages. Since in osteoarthritis (OA) both cartilage and subchondral bone suffer damages and WNT signaling pathway has a crucial role in their maintenance, the objective of the study was to analyze expression profile of RSPO family and its receptors [leucine-rich repeat-containing G-protein coupled receptors (LGRs)] in OA tissue samples as well as in differentiating chondrocytes and osteoblasts.

**Materials and Methods:**

In this experimental study, human early and advanced stage of OA tissue samples were analyzed for the morphological changes of articular cartilage by hematoxylin and eosin (H&E) staining, safranin-O staining and lubricin immunostaining. RSPOs and LGRs expression were confirmed by immunohistochemistry. Human primary chondrocytes and human osteoblast cell line, SaOS-2, were cultured in differentiation medium till day 14 and they were analyzed in terms of expression of RSPOs, LGRs and specific marker for chondrogenesis and osteogenesis by western blotting and quantitative reverse transcription polymerase chain reaction (qRT-PCR).

**Results:**

Advanced stage OA tissue samples showed increased expression of RSPO1 and LGR6 in a region close to subchondral bone. While RSPO2 and LGR5 expression were seen overlapping in the deep region of articular cartilage. Differentiating chondrocytes demonstrated elevated expression of *RSPO2* and *LGR5* from day 7 to day 14, whereas, osteoblasts undergoing differentiation showed enhanced expression of RSPO1 and LGR6 from day 2 to day 14. Under tumor necrosis factor alpha (TNFα) stimulatory conditions, RSPO2 and RSPO1 recovered the suppressed expression of inflammatory, chondrogenic and osteogenic markers, respectively. A recovery in the stability of β-catenin was also noticed in both cases.

**Conclusion:**

Spatial expression of RSPOs during progression of OA might be dynamically controlled by cartilage and subchondral bone. Interplay amid chondrocytes and osteoblasts, via RSPOs, might provide probable mechanisms for treating inflammatory pathogenic conditions like OA.

## Introduction

Degenerative osteoarthritis (OA) is hallmarked by synovial joints that suffer from degeneration of articular cartilage, causing alteration of cartilage structure and compositions along with changes in subchondral bone architecture (1, 2). Initially, it was proposed that alterations in bone take place secondary to cartilage degeneration, and they do not participate in the process of disease augmentation. Nevertheless, several animal studies have demonstrated that alterations in subchondral bone takes place at the initial stages of OA (3, 4), and that changes in subchondral bone can lead to degeneration of cartilage (5). The intimate physical interface amid cartilage and subchondral bone suggests biochemical and molecular interaction throughout this interface in healthy and osteoarthritic joints (6).

Amplified vascular communicating channels, microcracks and fissures throughout the interface and the asymmetrical bone cartilage anatomy could provide a transport conduit to assist molecular transport. During the OA progress, hydraulic conductance between articular cartilage and subchondral bone increases (7). Effector molecules produced from bone matrix metalloproteinase 2 (MMP2), receptor activator of nuclear factor κ-B ligand (RANKL), hepatocyte growth factor (HGF) or cartilage (i.e. interleukin 1; also known as IL1), metalloproteinases with thrombospondin motifs (ADAMTS) and MMP13 may crossover from one zone to another and they can alter the homeostasis of each other (8). Studies have confirmed that the nutrients from bone may nourish cartilage through the channels that links them, apart from the blood vessels (9, 10). In bovine explant cultures, chondrocyte survival is significantly influenced by subchondral bone (11). While, regulatory factors released from the chondrocytes in degraded cartilage might contribute to induction of osteoclastogenesis, and thus participate in the loss of subchondral bone during OA (12). Taken together, it may be proposed that interplay between the bone cartilage complexes is a holistic system, whereby multiple factors might contribute to OA pathogenesis.

Among various molecular regulators that affect cartilage and subchondral bone, WNT signaling pathway is crucial for maintaining the biochemical unit of bone and cartilage. Studies have demonstrated that both, inhibition or activation of canonical WNT signaling can have adverse effect on cartilage, including apoptosis of chondrocytes, perturb phenotype of articular chondrocytes, OA-like lesions, overexpression of markers related to hypertrophy and terminal differentiation (13, 14). While, activation of WNT signaling pathway, either by inhibiting WNT antagonists or increasing the stability of β-catenin, can have stimulatory effect on bone formation causing stiffer and thicker bones (15, 16). Since, various agonists and antagonists, which are often secretory in nature -like secreted frizzled-related protein (sFRP), sclerostin (SOST) and Dickkopf (DKK1)- tightly regulate WNT signaling, it is possible that bone and cartilage modulate each other via WNT signaling pathway and create pathological environment like arthritis. Overexpression of WNT signaling pathway agonists, WNT1-inducible signaling pathway protein1 (WISP1) and WNT16, has been described in human cartilage tissue samples after initiation of cartilage damage and synovium of OA (17, 18). Release of agonists can directly induce secretion of the aggrecanase and MMPs in chondrocytes, leading to destruction of cartilage. While, remodeling process in subchondral bone may be compelled toward bone formation resulting in development of osteophytes (19).

Rspondins (RSPOs) contain a thrombospondin type 1 domain/repeat-1 and they are cysteine-rich secretory proteins (20). In mammals, four members (RSPO1, RSPO2, RSPO3 and RSPO4) of RSPO protein family have been identified, having overall resemblance index of 40-60% in sequence homology and organization of domain (21, 22). In a high throughput sequencing study of human fetal brain cDNA library, RSPO3 was identified as the first member of the RSPO family (23). Thereafter, other members of RSPO family were identified from different species (20, 24, 25). To activate WNT signaling pathway, extracellular constituents of the WNT signaling acts synergistically with RSPOs (25-27). It has been observed that during developmental stages, expression of Wnt and RSPO proteins are either close or overlaps with each other, suggesting a probable relationship between RSPOs and WNT signaling pathway (28). Due to the capability of RSPOs to act as a regulator of WNT signaling pathway, several possible roles of these proteins have been suggested (27). Considering the functional role of RSPOs as agonists of WNT signaling, we tried to analyze expression pattern RSPOs along with its receptors [leucine-rich repeat-containing G-protein coupled receptors (LGRs)] during early and advanced stages of OA. An insight into the expression pattern of RSPOs and LGRs could be helpful in understanding the regulation of WNT signaling, as a cross-talk signaling mechanism between bone and cartilage during OA progression.

## Materials and Methods

### Histochemistry

In this experimental study, cartilage tissue samples from human femoral condyles were acquired from healthy patients (around 58- to 80-years old) undergoing surgery for hip replacements. The Ethical Committee of Hallym University-Sacred Heart Hospital, Chuncheon, South Korea (2009-42) reviewed the experimental procedure and granted permission. To examine the status of explanted cartilage damage, histochemical staining was performed on random samples of femoral condyles cartilage tissue pertaining to early and advanced OA stages. Explanted femoral heads were cleaned under sterilized conditions and harvested cartilage soft tissue was fixed by immersing in a solution of 2% paraformaldehyde (PFA, Merck, USA) for 24 hours. The samples were decalcified in 10% ethylenediaminetetraacetic acid solution (EDTA, SigmaAldrich, USA) before embedding in paraffin wax. Prior to staining, the tissues were deparaffinized and rehydrated.

### Hematoxylin and eosin staining

The paraffin-embedded samples were deparaffinized, rehydrated and 5 μm thick sectioned samples were cut. Representative sections from all cartilage subtype samples were stained with hematoxylin and eosin (H&E) for the descriptive analysis of morphological details. Light microscope at ×10 magnification (Ziess AxioCam digital camera, Carl Zeiss, Germany) was used to visualize and photograph the stained sections.

### Safranin-O staining

Safranin-O staining was carried out as follows. After steeping in Weigert’s iron hematoxylin solution for about 10 minutes, the samples were rinsed with normal alkaline tap water for 10 minutes. For 5 minutes, the samples were stained in fast green solution and bathed with 1% acetic acid for 10 seconds. Subsequently, 0.1% Safranin-O solution (Sigma-Aldrich, USA) was used to stain the samples for 5 minutes and they were dehydrated by using a graded series of alcohol. Next, the samples were cleared with xylene. Finally, each sample was mounted with resinous mounting medium for observation and image acquisition. The obtained results were visualized at ×10 magnification by a microscope and pictured by a Ziess Axi℃am digital camera.

### Immunohistochemistry

Lubricin, RSPOs and LGRs were immune stained using the two-step immunohistochemistry procedure according to the manufacturer’s protocol (Santa Cruz Biotechnology, USA). In short, the tissue sections were treated with rabbit polyclonal antibody for lubricin (Santa Cruz Immunohistochemistry Lubricin, RSPOs and LGRs were immune stained using the two-step immunohistochemistry procedure according to the manufacturer’s protocol (Santa Cruz Biotechnology, USA). In short, the tissue sections were treated with rabbit polyclonal antibody for lubricin (Santa Cruz Biotechnology, USA), RSPO1 (Sigma-Aldrich, USA), RSPO2 (Sigma-Aldrich, USA), LGR5 (Sigma-Aldrich, USA), LGR6 (Abcam, England) and β-catenin (Santa Cruz Biotechnology, USA) of 1:500 dilutions at 4˚C. The slides were washed thrice with phosphate-buffered saline (PBS, Wel Gene, Korea) and goat anti-rabbit immunoglobulin G (IgG, Santa Cruz Biotechnology, USA) was treated at room temperature for 30 minutes. Western blot bands were developed for visualization with 3, 3′-diaminobenzidine as the chromogen. Each section was photographed at ×10 magnification by a Zeiss Axio Cam digital camera.

### Preparation of primary human chondrocytes

Cartilage samples from human femoral condyles were dissected in 100 mm dish under sterilize environment. Samples were rinsed continually with Dulbecco’s Modified Eagle Medium (DMEM, Gibco, USA) including 10% fetal bovine serum (FBS, Gibco, USA) supplemented with 1% Penicillin-Streptomycin (P/S, Gibco). After digestion with Hyaluronidase in dish, sterilized blade was used to dissect the cartilage samples into small fragments. In serum free DMEM media, minced pieces of cartilage were washed twice and treated with protease buffer for one hour at 37˚C and 5% CO_2_. Again, in DMEM (serum free), the cartilage fragments were rinsed twice followed by enzymatic digestion with collagenase for nearly 2 hours and 30 minutes at similar condition as mentioned above. After completion of the enzymatic degradation, solution was filtered through cell strainer of 70 μm and stored in a 50 ml tube. Then, the media was centrifuged at a speed of 1500 rpm for 5 minutes and pellet so obtained was rinsed while performing the procedure twice. At the start, cell pellet was resuspended with complete DMEM (20% FBS and 1% P/S). Three days later, DMEM media (10% FBS and 1% P/S) was replaced to maintain the cells.

### Pellet culture of the primary human chondrocytes for differentiation

To induce chondrocyte differentiation, aliquots of 5×10^5^ cells were centrifuged at 250 g for 5 minutes (29). Then, chondrocyte cells were treated with 1X insulintransferrin-selenium x supplement premix (ITS-X, Gibco, USA). After 24 hours of incubation period, spherical aggregate of the sedimented cells were observed at the bottom of each tube. 1×10^5^ cells were grown in 60 mm dish for control. Primary chondrocytes were differentiated for 1, 7 and 14 days. The cells were cultured under optimal condition of 37˚C and 5% CO_2_. Once attached, the cells were cultured and medium was changed after every 3 days.

### Cultivation and differentiation of osteoblasts

SaOS-2 cells (Human osteosarcoma cell line, ATCC, HTB-85), were grown in complete DMEM (10% FBS and 1% P/S). To induce differentiation, osteoblasts were grown in osteogenic medium, containing 50 μg/ml ascorbic acid (Sigma-Aldrich, USA) and 10 mM β-glycerophosphate (Sigma-Aldrich, USA). 1×10^5^ cells per well were seeded in a 60 mm petri dish and grown at 37˚C and 5% CO_2_. After every 3 days of culturing in osteogenic medium, it was replaced. Osteoblasts were then differentiated for 1, 7 and 14 days.

### RNA isolation and quantitative reverse transcription polymerase chain reaction

As per the manufacturer’s guidelines, TRIzol reagent (Invitrogen, USA) was used to isolate entire RNA. First strand of cDNA was synthesized by using SuperScript ІІ (Invitrogen, USA) and 2 µg of total RNA. For each PCR mixture one-tenth of the cDNA was used in each quantitative reverse transcription PCR (qRT-PCR) supermix (EXPRESS SYBR green, BioPrince, Korea). qRT PCR was done by using a Rotor-Gene Q (Corbett RG3000, Australia). PCR reaction was accomplished by 50 cycles amplification at 95˚C for 20 seconds, 60˚C for 20 seconds and 72˚C for 25 seconds. Relative mRNA expression level of specific genes was standardized to *GAPDH* and quantified by using ΔΔCt method. The human PCR primer sequences utilized in the study are listed in Table 1.

**Table 1:** Human primer sequences for quantitative reverse transcription polymerase chain reaction


Gene	Primer sequence (5ˊ-3ˊ)

*RSPO1*	F: AGGCCTGCTTCAAGCCATAACTTCT
	R: GCTCATTTCACATTGCGCAGGACT
*RSPO2*	F: TGGCTCAGTGTGTGCTGAGAGAAT
	R: AAGGTCACGAGTGAGTAGCGCATT
*RSPO3*	F: TGCACTGTGAGGTCAGTGAATGGA
	R: AGGTTACCCTTTGCTGAAGGATGC
*RSPO4*	F: ACCACCAGTGACTTGAGCATCTGT
	R: TGATGGCAGAAGGATAGGCAGTGA
*LGR4*	F: TTGTGGGCAACTTCAAGCTG
	R: AACCCCAAAATGCACAGCAC
*LGR5*	F: TGTTTCAGTGGCCTGCATTC
	R: AAGGTCATGGCTTGCAATGC
*LGR6*	F: AACAACATCAAGGCCATCCC
	R: ATGCCGATCTTCCCACAAAC
*GAPDH*	F: TTCAGCTCAGGGATGACCTT
	R: ACCCAGAAGACTGTGGATGG


### Protein extraction and western blotting

The cells were instantly rinsed with PBS (ice-cold) after removing the media and incubated for 15 minutes with lysis cocktail buffer containing phosphatase and protease inhibitor (Roche Diagnostics, Germany). After centrifugation at 14,000 rpm for 15 minutes, the entire cell lysates were collected (separated from the cells debris). As per manufacturer’s protocol, protein assay kit (BioRad, USA) was used to determine protein amount in the samples. For each sample, equal amount of protein was loaded into 10% sodium dodecyl sulfate (SDS)-polyacrylamide gel followed by gel electrophoresis. Then, separated proteins were transferred to polyvinylidene fluoride (PVDF) membranes (Millipore, USA). The blots were incubated with 1:1000 dilutions of primary antibodies: RSPO1, RSPO2, LGR5 and LGR6, Col1α, Col2, osterix, IkBα, β-catenin, β-Actin (Santa Cruz Biotechnology, USA), Sox-9 (Abcam, USA) and Cox- 2 (Cell Signaling Technology, USA) in 1 % BSA. Blots were washed three times with TBST (10 mM Tris HCl, 50 mM NaCl, 0.25% Tween 20) and then treated with a horseradish peroxidase-conjugated secondary antibody (Jackson Immunoresearch, USA) followed by two times washing with TBST. Finally, the obtained bands were pictured by using chemiluminescence (ECL) reagents (BioNote Inc., Korea). Antibody against β-actin was considered as a loading control. Densitometric analyses of the western blots were also performed (Fusion FX, Vilver Lourmat, France).

### Statistical analysis

All of the statistical data were evaluated by Graphpad Prism 5.0 (GraphPad Software, USA) and assessed by two-tailed Student t test. Value of P<0.05, P<0.01 and P<0.001 was considered to designate statistical significance.

## Results

### Differential expression of RSPOs and LGRs at early and advanced stages of human OA samples

**Fig.1:**
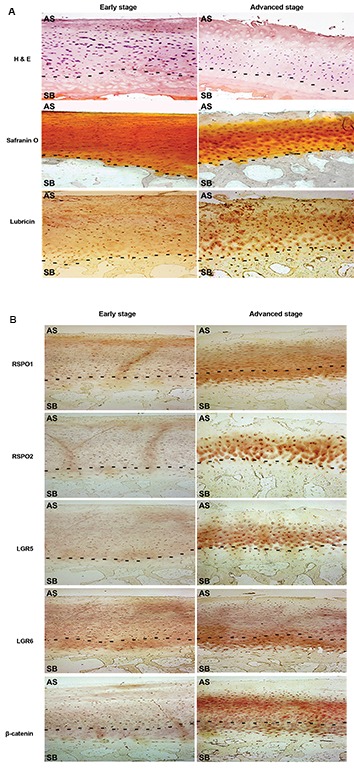
Expression of RSPOs, LGRs and β-catenin in human OA cartilage samples. **A.** H&E staining, safranin-O staining and IHC of lubricin in OA cartilage tissue samples with early and advanced stages obtained from femoral condyles cartilage. **B.** IHC showed that the expression of RSPOs, LGRs and β-catenin is significantly increased in advanced stage of OA cartilage compared to early stage. In these figures, above part of the dotted line represents articular cartilage whereas the lower part represents subchondral bone (approximate estimation, magnification ×10, scale bar: 100 µm). AS; Articular surface, SB; Subchondral bone, RSPOs; Rspondins, LGRs; Leucine-Rich Repeat-Containing G-Protein Coupled Receptors, OA; Osteoarthritis, and IHC; Immunohistochemistry.

To observe the pattern of expression of RSPO proteins and its receptors (LGRs) in OA tissue sample from human patients, tissue sections were categorized as early or advanced stage samples and they were immunostained for the proteins like lubricin, RSPOs, LGRs and β-catenin, as described in material and methods. H&E, lubricin and safranin-o staining demonstrated intact cartilage structure in the case of early stage OA samples, while a loss of articular surface, reduced expression of lubricin and decrease in width of cartilage was observed in advanced OA samples (Fig.1A). In advanced stage OA samples, spatial RSPO1 expression was observed close to subchondral bone plate which includes lower area of calcified cartilage and a thin cortical bone tissue layer (Fig.1B). Though, no localized expression of RSPO1 was observed in early stage OA samples. Expression of LGR6 appeared to overlap with the expression of RSPO1 (i.e. near the subchondral bone plate). The spatial expression of RSPO2 was very much localized towards the middle and deep zone of articular cartilage since upper layer of cartilage was found distorted in the advanced stage tissue samples. LGR5 expression was observed close to the area of expression of RSPO2 which is near to middle and deep zone of articular cartilage area. No expression of RSPO2 or LGR5 was visible in the early stage OA samples. Since, RSPOs has the ability to activate WNT signaling pathway, we evaluated expression level of β-catenin in early and advanced stage OA samples. In the advanced stage OA samples, expression level of β-catenin was increased around the overlapped region of RSPO1 and RSPO2.

### mRNA expression profile of RSPO proteins and its receptors during differentiation process of chondrocytes

**Fig.2:**
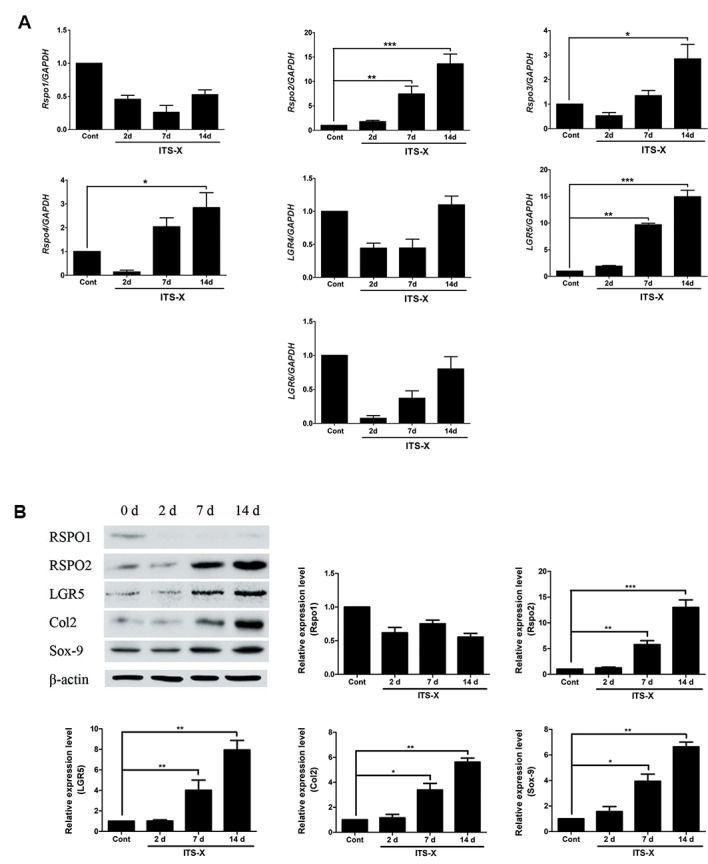
Expression of RSPOs and LGRs in human primary chondrocytes during differentiation. To induce differentiation, chondrocytes were treated with 1X ITS-X supplement. **A.** mRNA levels were measured by qRT-PCR after 2, 7 and 14 days of differentiation. Results are represented as fold-increase relative to GAPDH expression. **B.** Protein levels were measured by western blotting after 2, 7 and 14 days of differentiation. Quantitative densitometric analysis of protein was performed by using Fusion FX software. The results were normalized with β-actin expression. Data are shown as the mean ± SD of three independent experiments. *; P≤0.05, **; P≤0.01, ***; P≤0.001, RSPOs; Rspondins, LGRs; Leucine-Rich Repeat-Containing G-Protein Coupled Receptors, 1X ITS-X; Insulin-TransferrinSelenium-Ethanolamine, qRT-PCR; Quantitative reverse transcription polymerase chain reaction, Cont; Control, and d; Day.

Since RSPO2 showed increased expression level along with its receptor (LGR5) in advanced stages OA tissue samples, we tried to analyze the expression pattern of RSPO family proteins in differentiating chondrocytes. Initially, the primary chondrocytes were cultured as pellet culture and treated with InsulinTransferrin-Selenium-X supplement 1X (ITS-X) to induce differentiation for 14 days. mRNA was collected at days 2, 7 and 14 of the differentiation process of chondrocytes. qRT-PCR data displayed a substantial increase in the expression of *RSPO2* after 7 days (10 folds) of treatment which followed till day 14 (16 folds), while a small increase in expression pattern of *RSPO3* and *RSPO4* was observed at day 14 (3 folds) of differentiation process. In the case of RSPO receptors, *LGR5* demonstrated a significant increase in the mRNA expression level after 7 days (10 folds) of differentiation process followed till day 14 (15 folds). mRNA expression levels of other two receptors, namely *LGR4* and *LGR6*, did not show any significant change (Fig.2A). Findings obtained from western blot confirmed the mRNA expression results by demonstrating protein expression of RSPO2 in human primary chondrocytes pellet culture after 2 days of differentiation process till day 14. While, RSPO1 protein expression was not observed during this time. Protein expression of LGR5 was also observed to be enhanced during differentiation process of the chondrocytes (Fig.2B). An increment in the protein expression level of chondrogenic markers -like collagen (Col) 2 and master transcription factor, Sox-9 (sex-determining region Y-type high mobility group box protein; responsible for early chondrocyte differentiation) confirmed induction of differentiation process in the chondrocytes.

### mRNA expression profile of RSPO proteins and its receptors during differentiation process of osteoblasts

**Fig.3:**
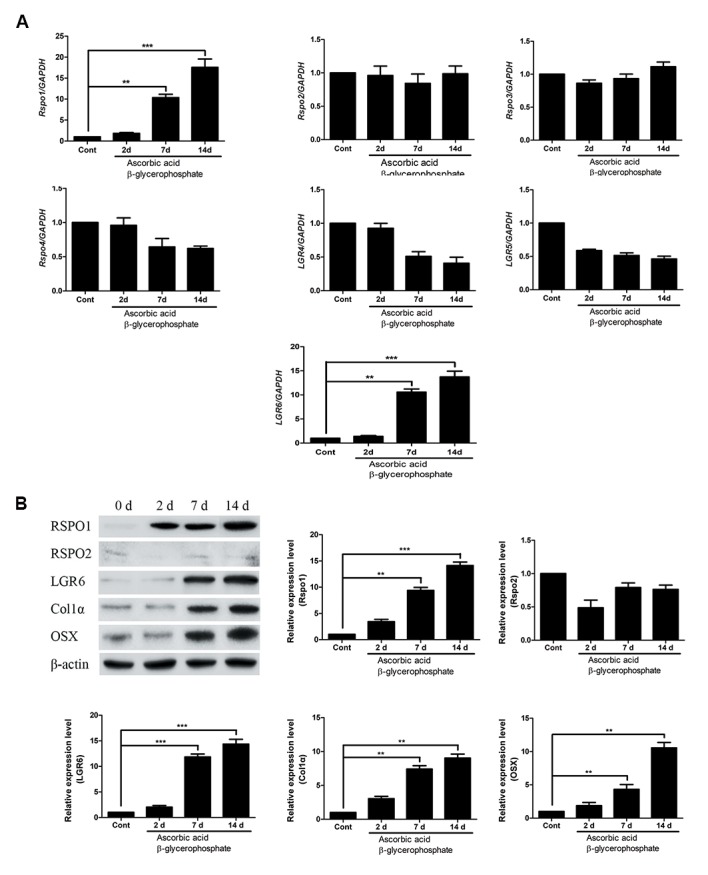
Expression of RSPOs and LGRs in osteoblasts during differentiation. To induce differentiation, osteoblasts were treated with 10 mM β-glycerophosphate and 50 µg/ml ascorbic acid. A. mRNA levels were measured by qRT-PCR after 2, 7 and 14 days of differentiation. Results are represented as fold-increase relative to GAPDH expression. B. Protein levels were measured by western blotting after 2, 7 and 14 days of differentiation. Quantitative densitometric analysis of protein was carried out by using Fusion FX software. The results were normalized with β-actin expression. Data are shown as the mean ± SD of three independent experiments. **; P≤0.01, ***; P≤0.001, RSPOs; Rspondins, LGRs; Leucine-Rich Repeat-Containing G-Protein Coupled Receptors, qRTPCR; Quantitative reverse transcription polymerase chain reaction, Cont; Control, and d; Day.

Osteoblasts are well known for differentiating into osteocytes and contributing to bone formation. This process is tightly regulated by several regulatory molecules like RSPOs. To observe the expression pattern of RSPOs during the process of osteoblast differentiation process, SaOS-2 cells were induced to differentiate by treating β-glycerophosphate (10 mM) and ascorbic acid (50 µg/ml). mRNA from SaOS-2 cells was collected after 2, 7 and 14 days of differentiation process. Expression level of RSPO1, RSPO2, RSPO3 and RSPO4 as well the receptors for *RSPOs, LGR4, LGR5* and *LGR6* were analyzed by qRT-PCR. Among RSPOs, mRNA expression level of RSPO1 was found to be significantly increased after 7 days (12 folds) of differentiation process till day 14 (20 folds). mRNA expression of the other RSPOs were not found to be significantly affected during the day 14 of differentiation. Among the receptors for RSPOs, *LGR6* was found to be elevated after 7 days (3 folds) of differentiation till day 14. *LGR4* and *LGR5* showed no significant alteration in mRNA expression level during differentiation process of osteoblasts (Fig.3A). In order to confirm the expression of RSPOs and its receptor at mRNA levels, we tried to analyze protein level of significantly expressed genes by western blotting. Western blot results demonstrated elevated protein expression of RSPO1 after 2 days of differentiation process in SaOS-2 cells, till day 14. However, protein expression of RSPO2 was not in accordance with the mRNA expression profile in osteoblasts during differentiation. Similar to mRNA expression of LGR6, western blot results also confirmed the expression of LGR6 after 7 days of differentiation process, till day 14. The process of differentiation in osteoblasts was confirmed with an increment in the protein level of osteogenic marker like Col1α and osterix (OSX, an osteoblast-specific transcription factor) from day 2 till 14 of differentiation process in osteoblasts (Fig.3B).

### Effect of RSPO2 during tumor necrosis factor alpha stimulatory conditions in chondrocytes

**Fig.4:**
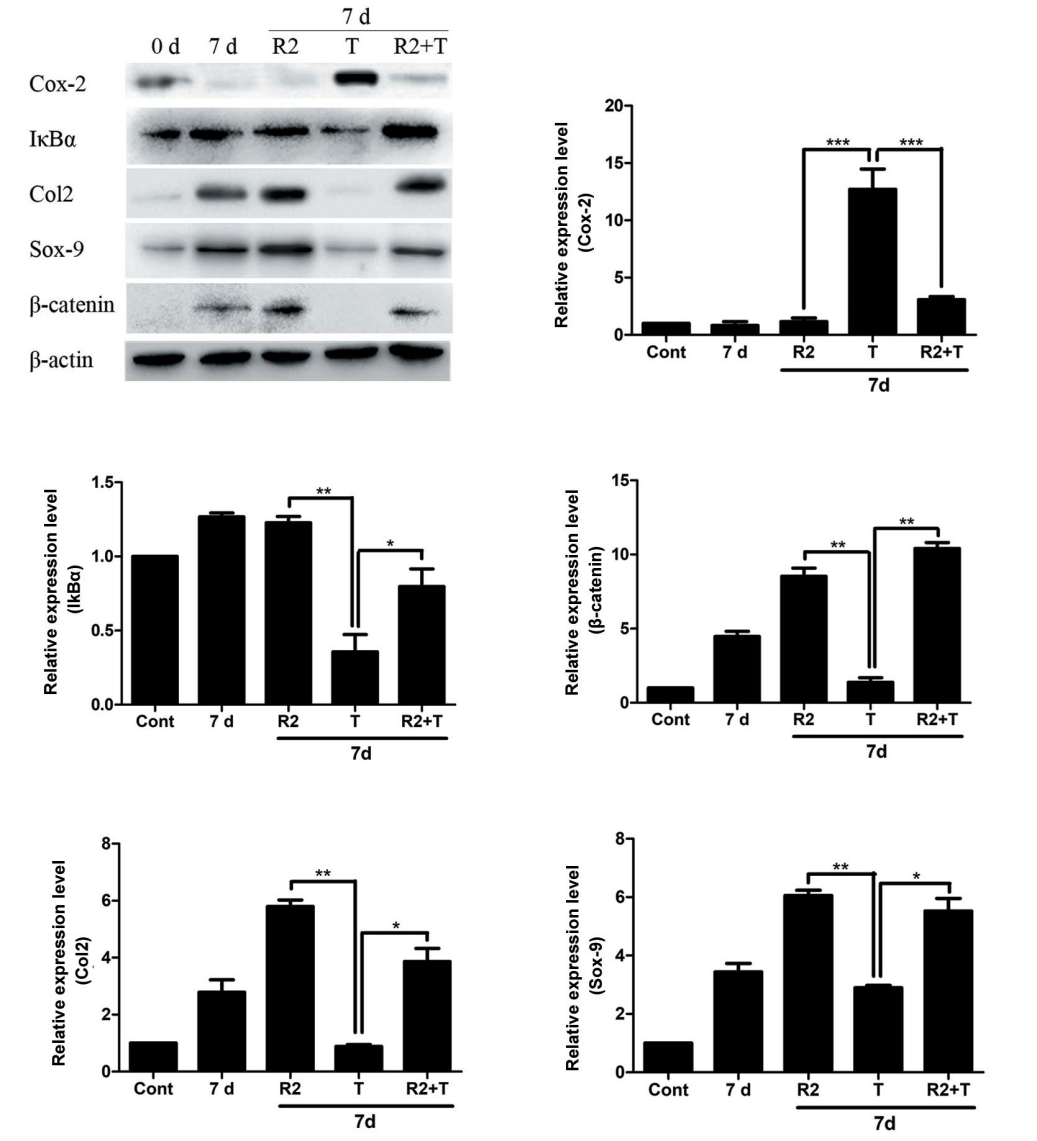
Effect of RSPO2 during chondrocyte differentiation under inflammatory conditions. Primary human chondrocytes were differentiated till day 7 by treating with 1X ITS-X. Thereafter, the cells were treated either with TNFα (T: 10 ng/ml) or along with RSPO2 (R2: 100 ng/ml) recombinant proteins. After 24 hours, protein expression levels of Cox-2, IkBα, Col2, Sox-9 and β-catenin were analyzed by western blotting. Protein expressions were detected by western blot. A relative densitometry analysis of protein bands was performed using Fusion FX software. The results were normalized with β-actin expression. Significant changes between the RSPO2 treated sample with TNF-α (alone) and TNFα with TNFα along with RSPO2 has been depicted. Data are shown as the mean ± SD of three independent experiments. *; P≤0.05, **; P≤0.01, ***; P≤0.001, RSPOs; Rspondin, TNFα; Tumor necrosis factor-alpha, Cont; Control, and d; Day.

Tumor necrosis factor alpha (TNFα) is a known inflammatory marker and is a major cytokine released during inflammatory pathological condition, like arthritis (30). To depict an *in vitro* inflammatory condition, TNFα (10 ng/ml) was induced to chondrocytes after 7 days of differentiation and the protein expression level of inflammatory marker like Cox-2 and stability of IκBα (NFκB signaling activation) were measured by western blotting (Fig.4). Moreover, to observe any effect by RSPO2 on the inflammation induced by TNFα, RSPO2 (100 ng/ml) was treated along with TNFα and expression levels of inflammatory marker were analyzed. Results demonstrated an increased protein level of Cox-2 and diminished IκBα stability in chondrocytes, indicating activation of NFκB signaling by TNFα, as expected. Interestingly, treatment of RSPO2 recovered the effect of TNFα and suppressed the Cox-2 expression, in addition to restoring the stability of IκBα. RSPO2 is known to induce WNT signaling activity and it was evident by the increased stability of β-catenin in chondrocytes. However, TNFα inhibited the β-catenin stability which was restored by the treatment of RSPO2. Effect of TNFα was also evident on the expression level chondrogenic markers, like Col2 and Sox-9, in differentiated chondrocytes which again was recovered after the treatment of RSPO2.

### Effect of RSPO1 during TNFα stimulatory conditions in osteoblasts

**Fig.5:**
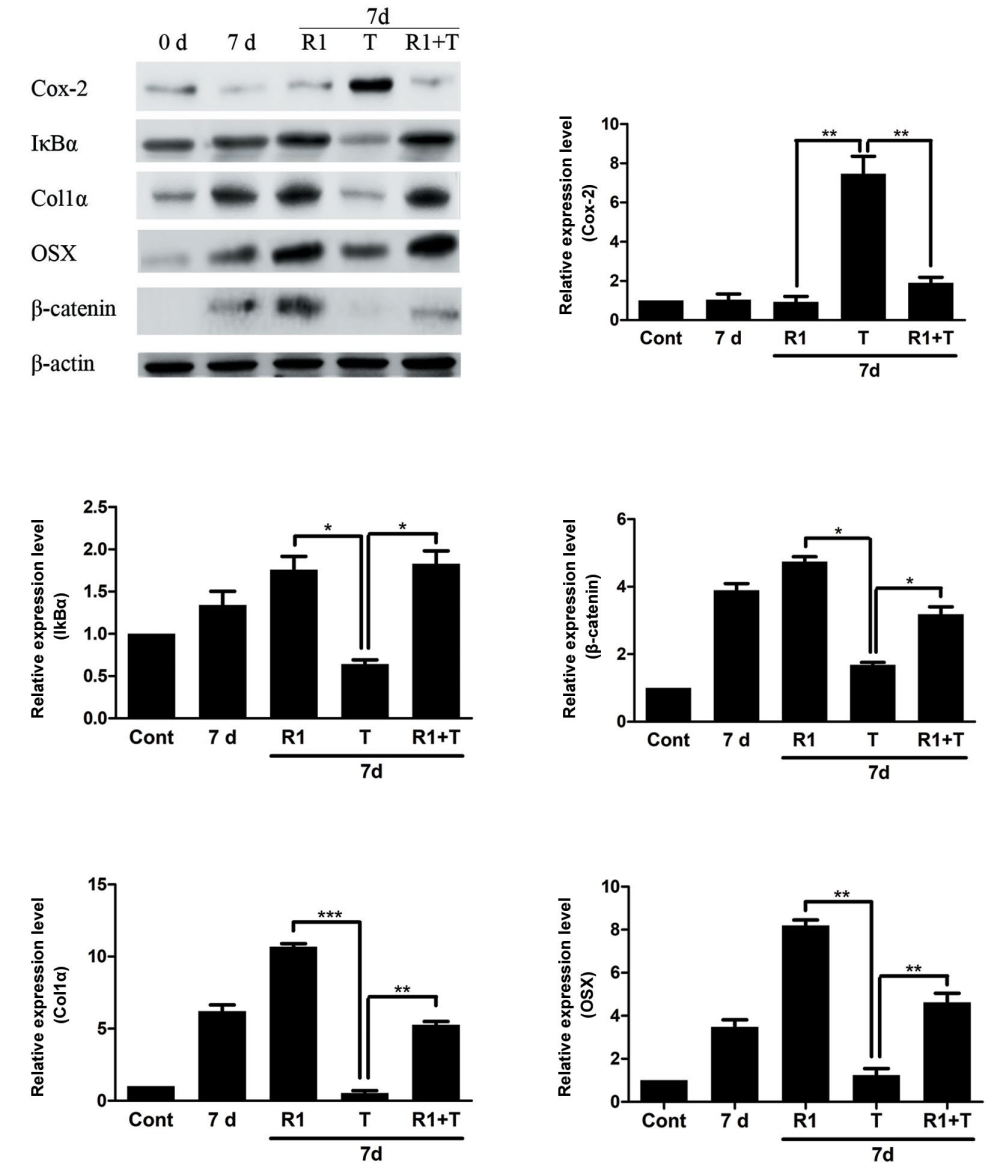
Effect of RSPO1 during osteoblast differentiation under inflammatory conditions. SaOS-2 cells were differentiated till day 7 by treating with 10 mM β-glycerophosphate and 50 µg/ml ascorbic acid. Then, SaOS-2 cells were treated either with TNFα (T: 10 ng/ml) or along with RSPO1 (R1: 100 ng/ml) recombinant proteins. After 24 hours, the protein expression levels of Cox-2, IkBα, Col1α, OSX and β-catenin were analyzed by western blotting. Protein expressions and a relative densitometry analysis were performed by using Fusion FX software. The results were normalized with β-actin expression. Significant changes between the RSPO1 treated sample with TNFα (alone) and TNFα with TNFα along with RSPO1 has been depicted. Data are shown as the mean ± SD of three independent experiments. *; P≤0.05, **; P≤0.01, ***; P≤0.001, TNFα; Tumor necrosis factor-alpha, Cont; Control, and d; Day.

Treatment of SaOS-2 cells with TNFα (10 ng/ml) 7 days after differentiation induced the expression of Cox-2 and decreased the stability of IκBα, implicating activation of NFκB signaling in SaOS-2 cells (Fig.5). The ability of RSPO1 (100 ng/ml) to induce WNT signaling activity was observed even in SaOS-2 cells as the protein level of β-catenin was found to be increased after RSPO1 treatment. Stimulation of RSPO1 to TNFα treated SaOS-2 cells decreased the protein levels of Cox-2, while it restored the stability of IκBα. Moreover, TNFα suppressed protein level of β-catenin, while it was recovered after the stimulation of RSPO1. As marker for differentiation process of osteoblasts, the protein levels of Col1α and OSX was increased in SaOS-2 cells after day 7 of differentiation process. However, treating with TNFα was able to suppress the protein levels of Col1α and OSX in 7 days differentiated SaOS-2 cells. Stimulation of RSPO1 to TNFα treated SaOS-2 cells restored the protein level of both Col1α and OSX.

## Discussion

OA is marked by a continual damage of articular cartilage accompanied with gradual loss of extracellular matrix, causing pain and functional disabilities in elder people (2). Regardless of extensive research efforts on OA, there is a massive need of effective therapies that can ultimately alter the natural course of this painful disease. With due efforts, recent researches have established that OA is not just a disease of articular cartilage, but the subchondral bone beneath. It also has a vital role in maintaining the health of the osteochondral unit (9). Studies focused on the molecular communications amid bone and cartilage interfaces might provide an understanding into the various mechanisms that control the vital molecular factors and signaling pathways involved in pathophysiology of OA (6, 19). Among the various factors that affects both cartilage and bone, WNT signaling pathway has been found to be activated during OA and it is thought to play critical role in tissue repair and fibrosis (31).

RSPOs are secretory proteins that have an ability to activate WNT signaling pathway and they are often co-expressed with WNTs (21, 27). During mouse development, RSPOs expression overlap with the expression of WNT signaling proteins, suggesting a likely association of RSPOs with the WNT signaling pathway (28). Rspo genes are differentially expressed during development of mouse limbs, implicating dynamic role of RSPOs during skeletal development (24, 28, 32). Recently, efforts were made to study the involvement of RSPO proteins in inflammatory arthritis animal model (TNFα transgenic mice) and it was demonstrated that RSPO1 was able to prevent bone and cartilage from inflammation-related damage (33). RSPO family proteins are dynamically expressed with distinct patterns during different mouse embryonic and fetal developmental stages (28). Henceforth, in order to understand the involvement of RSPO proteins in OA, we tried to analyze the expression pattern of RSPOs along with their receptors (LGRs) in early and advanced stage of human OA tissue samples. A progression based dynamic expression of RSPOs might explain its regulatory role during the pathogenesis of OA. Moreover, we tried to understand the pattern of expression of RSPO and LGR family during differentiation process of chondrocytes and osteoblasts, *in vitro*. In endochondral ossification, RSPO2 has been implicated to facilitate differentiation of chondrocytes by augmenting WNT signaling pathway (34). However, in animal OA models, increased stimulation of WNT/β- catenin signaling exerts hypertrophic differentiation in articular chondrocytes, which, in turn, results in enhanced expression of cartilage-degrading metalloproteinase and subsequent aggravation of OA. RSPO2 exerts this effect by binding to its receptor LGR5 (35). In agreement, expression profile of our results demonstrated an overlap between the expression of RSPO2 and LGR5 in advanced stage OA samples. The expression of RSPO2 and LGR was very much localized toward the deep region of articular cartilage. Moreover, *in vitro* data showed the expression of RSPO2 and LGR5 during differentiation process of chondrocytes. Here, it appears that expression of RSPO2 is critical for differentiation of chondrocytes and it is enhanced under pathological conditions, like OA the expression of RSPO2.

Previously, we have shown that RSPO1 can promote osteoblast differentiation process through WNT signaling pathway (36). Increased expression of LGR6 has been identified in the mesenchymal stem cells undergoing osteogenic induction and LGR6 has been suggested as an osteoblastic progenitor marker (37). In accordance to the above studies, we also observed that LGR6 expression overlapped with the expression of RSPO1 in advanced stage OA samples. In addition, the expressions of RSPO1 and LGR6 were detected during differentiation process of osteoblasts, implicating that LGR6 is possibly responsible for recognizing RSPO1 and mediating its effect for WNT signaling stimulation. However, further experiments are needed to ascertain this fact.

Numerous studies have indicated that TNFα plays a critical role, not only during the pathogenesis of inflammatory arthritis but also during degenerative joint disease like OA (30, 38). TNFα is responsible for maintaining the homoeostasis of matrix synthesis and its degeneration in articular cartilage of tandem with other cytokines like IL1, transforming growth factor β. Moreover, TNFα role has been shown in induction of bone loss during inflammatory conditions by affecting WNT signaling pathway (39, 40). In order to mimic the pathological conditions that might prevail during OA, we simply stimulated the chondrocytes and osteoblasts with TNFα and induced inflammatory response in these cells. Interestingly, co-treatment of TNFα along with RSPO2 in chondrocytes and RSPO1 in osteoblasts not only recovered the induction of inflammatory marker like Cox- 2, but also suppressed activated NFκB signaling in both of the cell types. Moreover, TNFα, suppressed chondrogenic markers (Col2 and Sox-9) and osteogenic markers (Col1α and OSX), were found to be recovered after co-treatment with RSPO2 and RSPO1, respectively. Additionally, TNFα, suppressing β-catenin stability, was restored by treatment of RSPO2 and RSPO1 in chondrocytes and osteoblasts, respectively. These results point towards a regulatory role of RSPOs in inflammation which might be achieved by activating WNT signaling pathway. TNFα has been shown to induce secretion of WNT antagonists, like DDK1 and SOST, from differentiating osteoblasts affecting their bone forming ability (39). Moreover, the localized expression pattern of RSPO1 (near to subchondral bone area) and RSPO2 (near to deep articular cartilage area) in the OA samples raise a possibility of interplay between chondrocytes and osteoblasts. Though, it appears to be interesting, further studies would be needed to delineate the mechanism by which WNT signaling pathway might interact with the inflammatory mechanism under the regulation of RSPOs. For example, further studies focused on the release of WNT signaling antagonists, in response to TNFα in chondrocytes and osteoblasts and finding any role of RSPOs in regulating this process would be quite interesting. Limitation of our study is that we have just considered TNFα as a stimulator for inflammation *in vitro*, while inflammation during OA pathogenesis is a multifactorial event, involving a diverse kind of pro-inflammatory factors and cytokines. For instance, other than TNFα, IL1β is the other cytokine that affects both chondrocytes and osteoblasts in joints. Hence, future studies should try to reveal the effect and role of IL1β or a combination of other cytokines in presence of RSPOs. A clear understanding of RSPOs antiinflammatory role under inflammatory conditions, like OA, would be helpful to suggest novel therapeutic agents in near future.

## Conclusion

During pathogenesis of OA, both articular cartilage and subchondral bone shows morphological and biochemical changes. OA does not simply represent an event of wear and tear process, but instead it is an atypical remodeling process leading to joint failure. An intermolecular interaction between articular cartilage and subchondral bone interface is being regarded as the contributing factor for altered structural and functional characteristics of this unit. RSPO family of proteins is known to stimulate WNT signaling pathway. Chondrocytes and osteoblasts need functional role of WNT signaling pathway during their developmental process as well as in pathogenic state. Thus, as key molecules for WNT signaling pathway, RSPOs might play a crucial role during their cross-talk based on their differential expression patterns. Our results in OA tissue samples demonstrate spatial expression of RSPO1 and RSPO2 along with their receptors, respectively LGR6 and LGR5, in early and advanced stage of OA samples. *In vitro* differentiation analysis of chondrocytes and osteoblasts also demonstrated correlation of expression pattern of RSPOs along with its receptors. Interestingly, the ability of RSPOs to recover adverse effect induced by TNFα represents possible role of RSPOs in affecting inflammatory pathways through WNT signaling. However, more detailed studies would be required to ascertain the functional role of RSPOs during inflammation. In brief, RSPOs might be the regulatory molecule and they may explain the relationship amongst cartilage and subchondral bone under pathogenic conditions like OA. A clear insight into the differential expression of RSPOs and their functional role might contribute to identify novel therapeutic targets for the cure of OA in near future.
